# Electro-spray deposited TiO_2_ bilayer films and their recyclable photocatalytic self-cleaning strategy

**DOI:** 10.1038/s41598-022-05633-w

**Published:** 2022-01-28

**Authors:** Kewei Song, Yue Cui, Liang Liu, Boyang Chen, Kayo Hirose, Md. Shahiduzzaman, Shinjiro Umezu

**Affiliations:** 1 Graduate School of Creative Science and Engineering, Department of Modern Mechanical Engineering, Waseda University, 3-4-1 Okubo, Shinjuku-ku, Tokyo, 169-8555 Japan; 2 Anesthesiology and Pain Relief Center, The University of Tokyo Hospital, 7-3-1 Hongo, Bunkyo-ku, Tokyo, 113-8655 Japan; 3 Nanomaterials Research Institute, Kanazawa University, Kakuma, Kanazawa, 920-1192 Japan; 4 Department of Modern Mechanical Engineering, Waseda University, 3-4-1 Okubo, Shinjuku-ku, Tokyo, 169-8555 Japan

**Keywords:** Materials for energy and catalysis, Chemistry

## Abstract

Recyclable titanium dioxide (TiO_2_)-based photocatalytic self-cleaning films (SCFs) having a bilayer structure were prepared and assessed. These SCFs comprised two layers of fibers fabricated using an electrospinning process. The self-cleaning layer was made of acrylonitrile–butadiene–styrene (ABS) fibers with embedded TiO_2_ while the substrate layer was composed of fibers made by simultaneously electrospinning poly (vinyl alcohol) (PVA) and ABS. This substrate improved the mechanical strength of the SCF and provided greater adhesion due to the presence of the PVA. The experimental results showed that the hydrophobicity (as assessed by the water contact angle), photocatalytic properties and self-cleaning efficiency of the SCF were all enhanced with increasing TiO_2_ content in the ABS/TiO_2_ fibers. In addition, the introduction of the substrate layer allowed the SCFs to be applied to various surfaces and then peeled off when desired. The ABS fibers effectively improved the strength of the overall film, while deterioration of the ABS upon exposure to UV light was alleviated by the addition of TiO_2_. These SCFs can potentially be recycled after use in various environments, and therefore have applications in the fields of environmental protection and medical science.

## Introduction

Recently, the challenges of resource exhaustion and environmental pollution have become of increasing concern worldwide. Taking air pollution as an example, the concentration of fine particles in the atmosphere has been an issue for several years. According to the World Health Organization Air Quality Guidelines, the concentration of PM2.5 in 95% of the countries and regions in the world exceeds the specified concentration threshold of 10 μm/m^3^^1^. Pollutants of this type can also have negative effects in terms of aesthetics, because they are readily adsorbed on the surfaces of buildings and other structures, affecting their appearance. Various other atmospheric pollutants such as nitrogen oxides can also produce acid rain, which has numerous deleterious effects on external structures.

Self-cleaning materials (SCMs) have received attention as a means of addressing these issues. These substances are typically applied to the surfaces of various materials to remove contaminants that have adhered as a consequence of exposure to pollutants and/or the elements (such as wind and rain). The range of potential applications for SCMs has become extensive, expanding from the original uses in the construction coating industry to emerging applications in automobiles, greenhouses, electronic equipment, and the medical and health fields. Thus, SCMs are currently regarded as one of the most promising so-called green materials^2–7^.

There are two main types of SCMs, depending on the self-cleaning principle and preparation process. One type is based on the preparation of superhydrophobic surfaces with low surface energy chemical compositions and microstructural roughness^8–11^. These surfaces remove dirt by repelling water droplets, through a phenomenon known as the “lotus effect”^12–17^. The second class of SCMs applied to surfaces are those that take advantage of the photocatalytic activity of inorganic semiconductor materials, such as TiO_2_. These materials degrade organic matter such as low molecular weight organic pollutants adsorbed on surfaces to generate carbon dioxide (CO_2_), water (H_2_O) and inorganic compounds^18–25^. This method avoids the disadvantages of the former type of SCMs, such as complex preparation processes, difficult scale-up and short service life, and thus has more potential for real-world applications.

TiO_2_ nanoparticles are the most promising photocatalysts for this purpose^26–30^ because they are both inexpensive and non-toxic, and can also serve as efficient electron transport layers in perovskite-based solar cells^31–35^. Consequently, SCMs in the form of films and coatings based on TiO_2_ nanoparticles have been widely used in many fields, such as wastewater treatment^36–39^, air purification^40,41^, chemical synthesis^42,43^ and electrode fabrication^44^.

Electro-spraying is a bottom-up fabrication strategy that has proven to be a very promising means of achieving high-resolution printing. In this process, a highly viscous liquid containing a solid such as TiO_2_ is discharged in the form of a spray via an electrostatic force in the direction perpendicular to a substrate such as fluorine-doped tin oxide, without the need for a vacuum environment^45^. Compared with other bottom-up techniques, electro-spray offers a more cost-effective and simpler approach to obtaining high-quality TiO_2_ films^46^. The film thickness is also readily controlled and the film can cover large areas with high reproducibility. The fabrication of photocatalytic hydrophilic self-cleaning films (SCFs) using electrostatic spinning process with TiO_2_ nanoparticles as photocatalytic substances therefore shows promise. Even so, although many different SCFs have been devised, challenges remain in terms of improving the mechanical strength, surface wettability, service life, and substrate adhesion and stability^47,48^. In this context, composite materials could be advantageous because such materials maintain the performance advantages of the individual components but can also provide synergistic effects that allow a wider range of applications^49–51^. Thus, the current issues related to SCFs could be addressed by innovations in the SCF components and preparation processes.

The present study prepared a reusable hydrophilic SCF having a bilayer structure. Acrylonitrile–butadiene–styrene (ABS) resins exhibit exceptional toughness and mechanical strength^52,53^. Thus, this work employed SCF substrates made of fibers comprising a blend of ABS and poly (vinyl alcohol) (PVA) fabricated by simultaneous spinning. These substrates provided good strength and adhesion properties^54^, allowing the SCFs to be reused. In these structures, an ABS/TiO_2_ fiber film prepared on the substrate layer provided the self-cleaning ability. The microstructures and adhesion of films having different TiO_2_ concentrations were compared and the self-cleaning efficiencies of these materials were examined. In addition, the reuse of the substrates was assessed, as well as the ability of the TiO_2_ to inhibit photodegradation of the ABS. These experiments demonstrated the advantages of the proposed double-layer SCF structure, which provides improved strength, stability and service life, and demonstrates a new approach to the preparation of functional materials with a wide range of applications.

## Experimental

### Materials

*N*,*N*-Dimethylformamide (DMF) was purchased from Hayashi Pure Chemical Industries, Ltd., Japan. ABS resin (FES-175ABS-1000-WH) was obtained from ABEE, Ltd., Japan. The TiO_2_ used in this work was Anastasi ST-01, purchased from Ishihara Sangyo Kaisha, Ltd., having an average particle diameter of 7 nm. The PVA had a molecular weight of 146,000‒186,000 and was purchased from the Aldrich Corporation. Ultrapure water was used in all experiments. All other reagents were analytical grade and were used without purification.

### Synthesis of materials

A 10 g quantity of the ABS powder was dissolved in 30 g DMF to prepare a 25 wt% solution. A separate series of 25 wt% ABS solutions in DMF were made and TiO_2_ nanoparticles were added to these to make a series having TiO_2_ proportions (relative to the combined TiO_2_ and ABS mass) of 0, 5, 10, 15, 20, 25, 30, 35 and 40 wt%. Following this, 3 g PVA was dissolved in 27 g DMF to prepare a 10 wt% PVA solution. Each of the above solutions was heated in a 60 °C water bath with vigorous stirring and then further heated at 60 °C in an oven for 24 h to ensure that the polymers were completely dissolved. The resulting mixtures are referred to herein as the ABS electrospinning solution (ABS-ESS), ABS/TiO_2_ ESS (ABS/TiO_2_-ESS) and PVA ESS (PVA-ESS), respectively.

### Preparation of the SCF samples

The ABS-ESS and PVA-ESS were added to two separate 20 ml syringes installed on microinjection devices situated on both sides of the receiving drum. The two nozzles used for injection in this device were connected to the positive pole of the high-voltage power supply and the end of the drum was wrapped with aluminum foil and grounded to act as the receiving end. The PVA and ABS solutions were passed through the two electrospinning nozzles simultaneously to form the substrate film using a voltage of 12.5 kV, 23G nozzle tips, a 23.4 μm/min advancing rate, a 100 mm receiving distance, a drum rotation rate of 300 rpm, spin time of 6 h, temperature of 26 °C and humidity of 35%.

After spinning for 6 h, a blended ABS/PVA fiber film was obtained for use as the SCF substrate layer. At this point, the two spinning solutions were replaced with a syringe filled with the TiO_2_/ABS mixture, and the same spinning conditions were employed to fabricate the self-cleaning upper layer on the blended film. Following electrospinning of the upper layer, the composite film specimen composed of a TiO_2_/ABS upper layer and an ABS/PVA lower layer was carefully removed from the aluminum foil. Video S1 shows the preparation process.

### Characterization and experiment

Representative SCF samples were coated with gold and the morphologies of the ABS/TiO_2_ nanofibers were investigated by scanning electron microscopy (SEM; JSM-5300, JEOL Ltd., Tokyo, Japan), operating at 10 kV. BEC-T mode of SEM equipment is used to observe the TiO_2_ particles in the film. The porosity of each SCF was assessed using the n-butanol uptake method. In this process, the dry mass of the film (w) was determined, after which the sample was immersed in n-butanol for 2 h. The SCF surface was subsequently dried with filter paper and its wet mass (w2) was found. The porosity was then calculated as P = (w2 − w1)/ρv, where ρ is the density of n-butanol and v is the volume of the film before wetting. Water contact angle measurements were performed to study the wetting behaviors of the SCFs. X-ray diffraction analysis (XRD; Miniflex, RIGAKU Ltd., Tokyo, Japan) was used to determine the crystallographic structure of the SCF. UV–Vis absorption spectra of nanofiber films with different components were measured. The self-cleaning efficiency of different kinds of nano TiO_2_ was compared. The self-cleaning efficiency of each specimen was examined by monitoring the photocatalytic activity of the material in response to UV-A, UV-B and UV-C radiation (fl6blb/N, Toshiba; gl6e, Sankyo Electric; CTUV-6, Coospider). UV–Vis spectrophotometry was used to determine the photocatalytic activities of the SCFs. In these trials, 1 × 1 cm specimens of ABS/TiO_2_ composite films containing from 0 to 40 wt% TiO_2_ were placed in beakers to which 100 ml portions of a 5 mg/l methylene blue solution were added. Each solution was then stirred for 30 min to achieve an equilibrium between the adsorption and desorption of the dye on the surface of the photocatalyst. Following this, the absorbance at the maximum absorption wavelength of methylene blue (465 nm) was determined using a UV–Visible spectrophotometer 4 h later and the concentration of the dye was calculated. The effect of TiO_2_ concentration on the photocatalytic rate was studied. Experiments on the recyclability of SCFs with different TiO_2_ concentrations were carried out indoors. At first, films of different TiO_2_ concentrations (0%, 5%, 10%, ~ 40%) were dropped with organic pollutant (methylene blue) and placed under a UV lamp (Wavelength: 315–400 nm). Then, the self-cleaning process was recorded every two hours until completing. After the film was peeled off and attached to other locations (Contamination point 2), the above experiment was repeated to verify the reusability of the films. The adhesive strengths of the SCFs were determined using the force tester (MCT-2150, AND Ltd., Tokyo, Japan). The reduced photodegradation of the ABS after mixing with the TiO_2_ was examined by acquiring Fourier transform infrared (FTIR) spectra of films before and after exposure to light (FT/IR-4200, Japan Spectroscopy).

## Results and discussion

### Double-layer structure self-cleaning film

**Figure 1:**
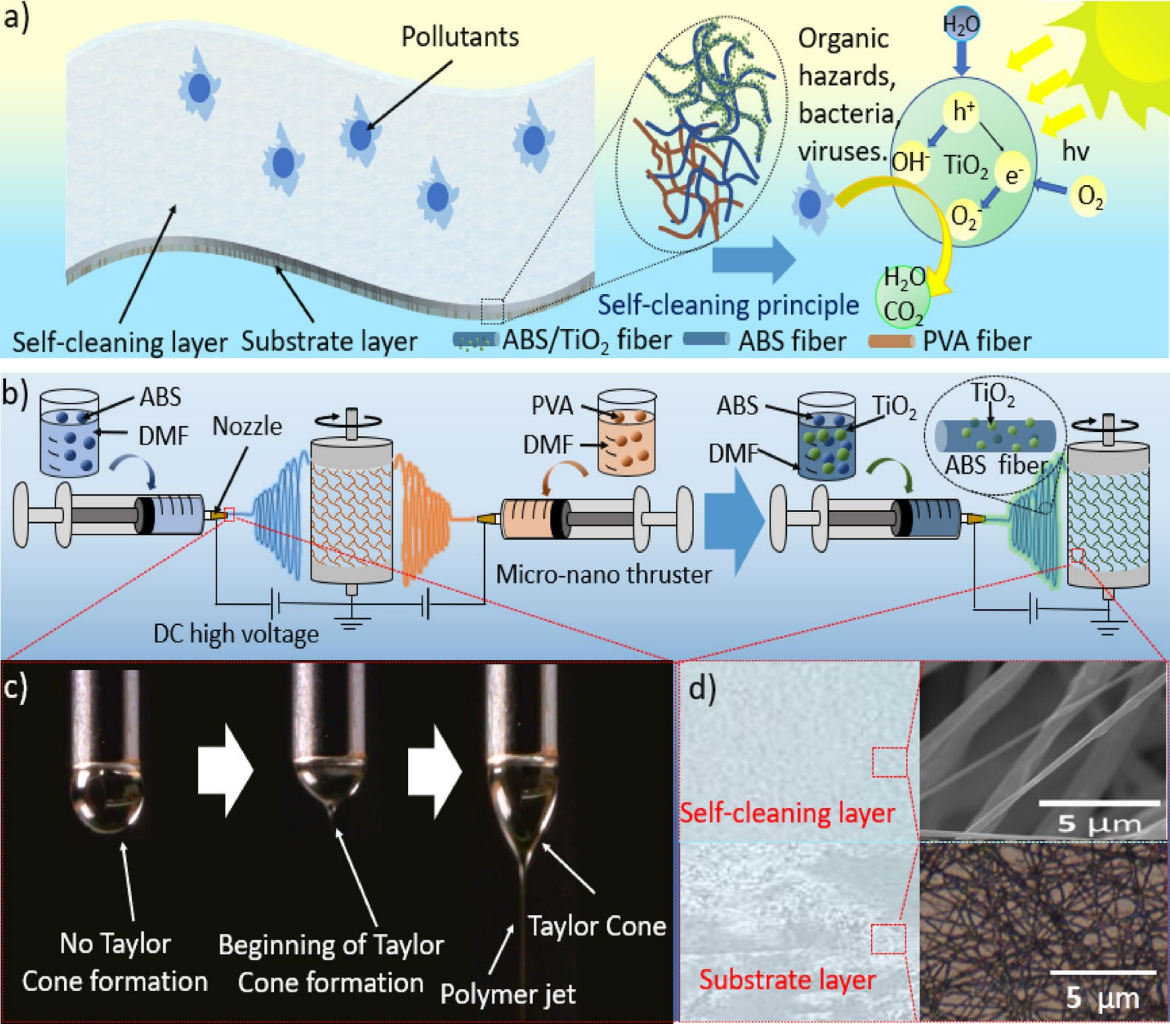
(**a**) The diagram showing the structural characteristics of the proposed SCF and its self-cleaning principle. (**b**) The diagram showing preparation of the SCF via a double nozzle electrospinning process. (**c**) Photographic images of the formation of a Taylor cone in this study. (**d**) SEM images showing the structural characteristics of the SCF.

As noted, the SCF prepared in this work had a double-layer structure (Fig. 1a) with an upper self-cleaning layer consisting of ABS fibers having embedded TiO_2_ nanoparticles. The underlying substrate layer comprised a mixture of ABS and PVA fibers that provided mechanical strength to the SCF and allowed the material to be applied to various surfaces and later peeled off. Figure 1b details the process of obtaining the proposed bilayer self-cleaning film using the electrostatic spinning process. The process was adjusted (Change from dual nozzle to single nozzle) after the substrate layer film was first obtained by simultaneous electrospinning with two nozzles, thus realizing the preparation of ABS/TiO_2_ fiber film. The concentrations of the solutions used in the electrospinning process were adjusted to obtain moderate adhesion suitable for this technique. Figure 1c shows the formation of a Taylor cone during an electrospinning trial, while the SEM images in Fig. 1d present the structural characteristics of the double layer SCF.

### Microstructure of TiO_2_ nanofibers characterization

**Figure 2:**
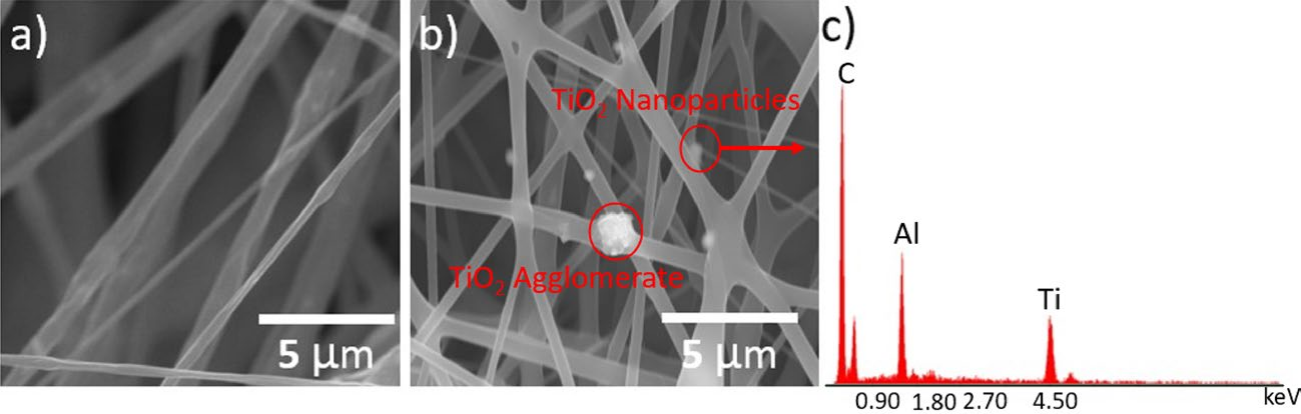
(**a**) SEM images of pure ABS fiber film. (**b**) ABS/TiO_2_ composite fibers in BEC-T mode. (**c**) Determination of Ti element in ABS/TiO_2_ composite fiber film by EDS.

**Figure 3:**
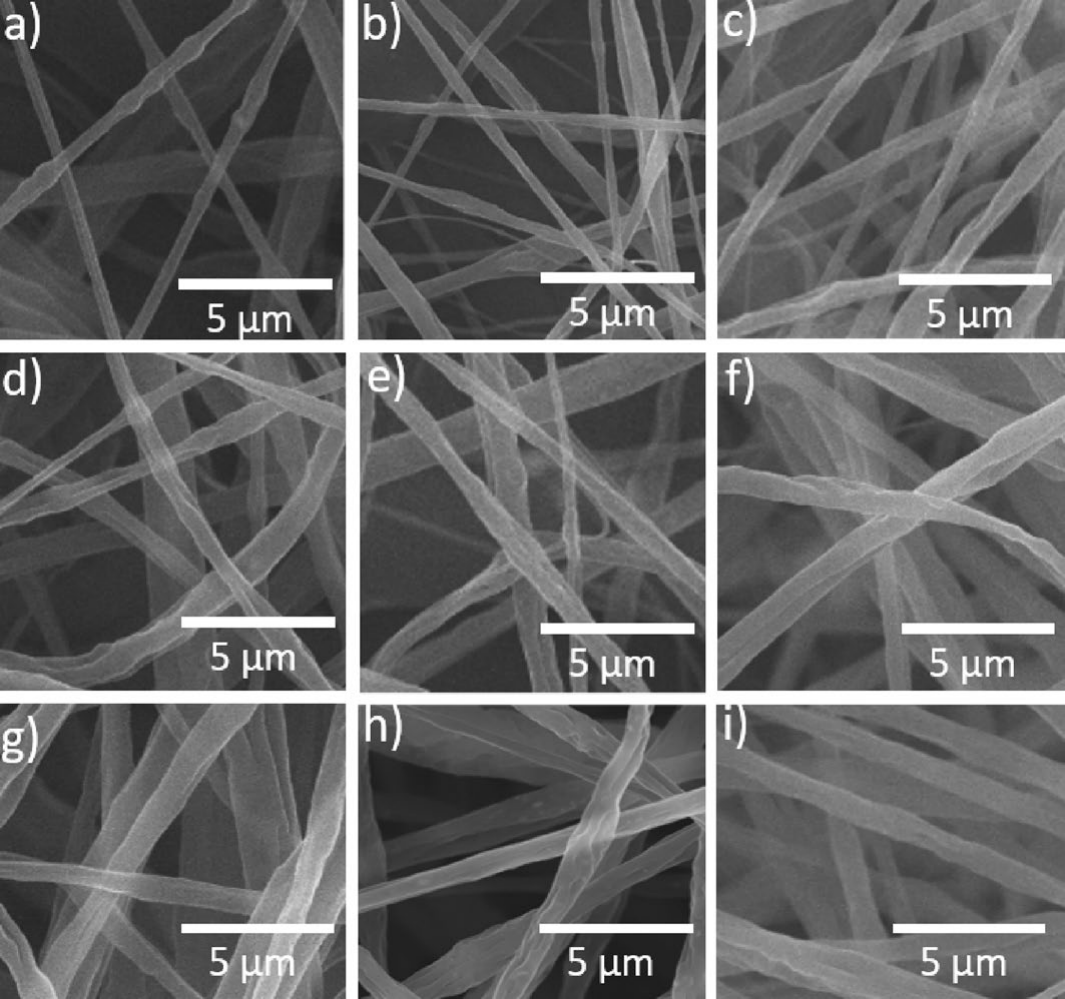
SEM images of SCF samples with different TiO_2_ concentrations showing the surface microstructures of ABS/TiO_2_ fibers with a TiO_2_ concentration of (**a**) 0, (**b**) 5, (**c**) 10, (**d**) 15, (**e**) 20, (**f**) 25, (**g**) 30, (**h**) 35 and (**i**) 40 wt%.

**Figure 4:**
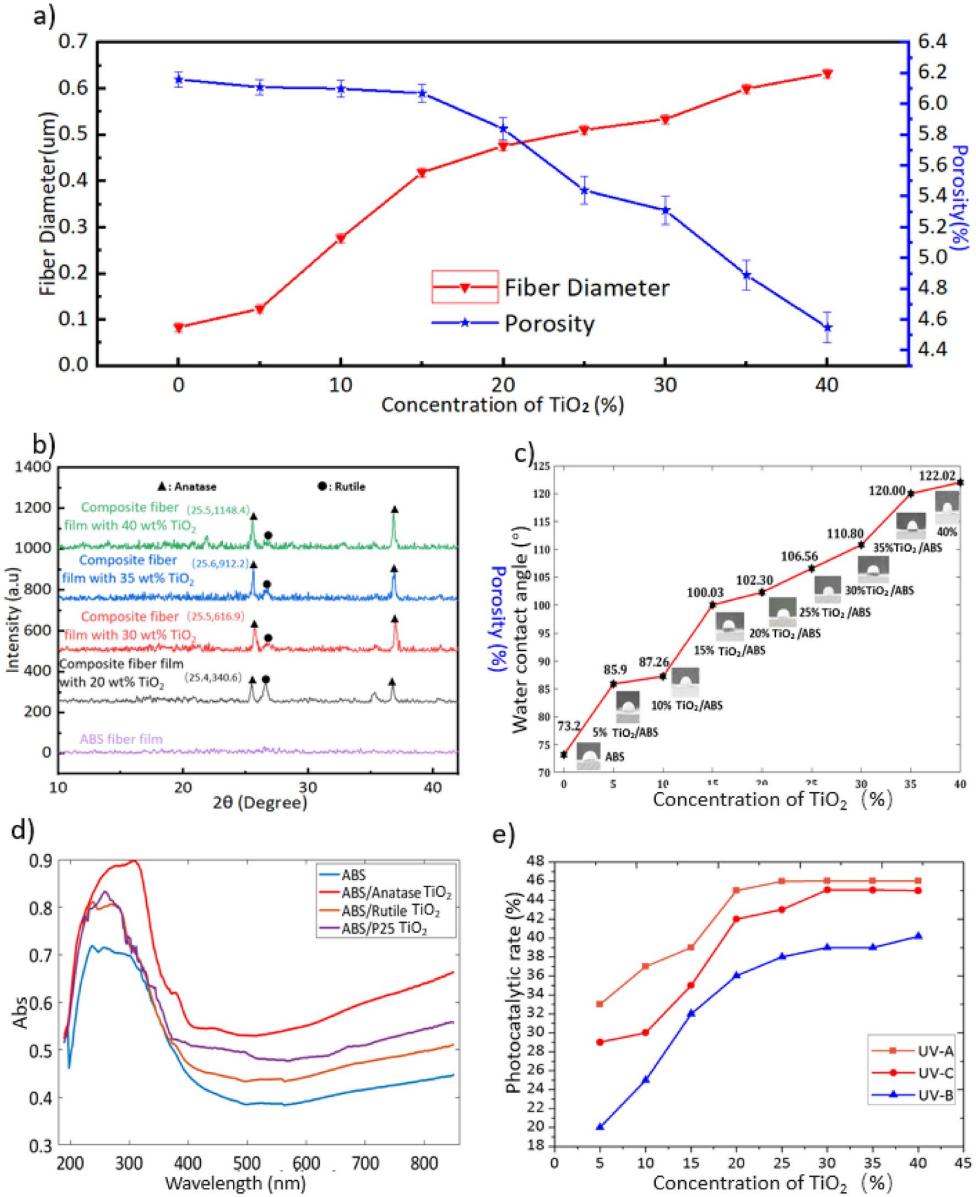
(**a**) The effect of TiO_2_ concentration in ABS/TiO_2_ on the surface microstructure (fiber diameter and porosity) of SCFs. (**b**) XRD curve of ABS fiber film, composite fiber film with 20 wt%, 30 wt%, 35 wt% and 40 wt% TiO_2_. (**c**) Wetting behavior of SCFs. (**d**) UV–Vis absorption spectra of nanofiber films with different components. (**e**) The effect of TiO_2_ concentration on the photocatalytic rate.

SEM images were acquired to assess the surfaces of the nanofibers in these samples, as shown in Fig. 2. Comparing the SEM image (Fig. 2a) and BEC-T image (Fig. 2b) of pure ABS fiber and ABS/TiO_2_ fibers indicates that the TiO_2_ particles were dispersed the fibers. In some parts of the film, a small amount of “aggregates” were observed, which are several times larger than TiO_2_ in size particles (Fig. 2b). This may be caused by the large aggregates of TiO_2_ due to the incomplete dispersion of a small amount of TiO_2_ in the ABS. The energy dispersive X-ray spectroscopy (EDS) experiment confirmed the existence of these TiO_2_ (Fig. 2c). It is also evident that the blending of these nanoparticles at relatively low concentrations did not affect the surface morphology of the ABS nanofibers. However, the nanoparticles did modify the nanofiber size, such that increased TiO_2_ levels increased the nanofiber size (see Fig. 3). Figure 4a plots the fiber diameters and porosities of the SCF specimens as functions of the TiO_2_ concentration. After mixing 5 wt% TiO_2_, the nanofibers diameters increased from 84 nm (Pure ABS fiber) to 124 nm. The fibers were found to range in diameter from 84 nm (Pure ABS fiber) to 633 nm (Composite fiber with TiO_2_ of 40 wt%), while the porosity varied from 6.16 to 4.55%. The data show that the diameter values exhibited a positive correlation with the concentration of TiO_2_ but the porosity decreased as more TiO_2_ was added. The increase in fiber diameter can be attributed to the increase of the solution viscosity induced by inclusion of TiO_2_ in the electrospinning solution. With the increase of TiO_2_ concentrations, the surface tension of solution was consequently enhanced which also increased the difficulty in the formation and stretching of solution jets.

### X-ray diffraction study

To investigate the crystalline structure of supposed films, X-ray diffraction (XRD) spectra were studied, as shown in Fig. 4b. It was found that the crystallinity of the ABS nanofibers was affected by blending the nanoparticles of TiO_2_. The XRD patterns of the pure ABS nanofiber film did not exhibit any obvious diffraction peaks. The diffractogram of AB/TiO_2_ sample exhibited three strong diffraction peaks at 25°, 27° and 38°, which correspond to the (101) and (004) crystal planes of anatase and (110) crystal planes of rutile^55^. This indicates the crystalline phase of TiO_2_ in this sample in which both anatase and rutile types exist. when the concentrations of TiO_2_ increased from 0 to 40 wt%, the anatase peaks intensity increased simultaneously. With the addition of TiO_2_ (from 0 to 20 wt%), the anatase peak began to appear. With the increase of TiO_2_ concentration (from 20 to 35 wt%), the anatase peaks showed a slow increase trend overall. When the TiO_2_ concentration is 40 wt%, the peak value showed lower than 35 wt%. We concluded that the anatase peaks are stronger as the TiO_2_ concentration increases. The above trend shows that the addition of TiO_2_ and the increase in concentration may affect the crystal structure to a certain extent due to cross-linking^56^.

### Water contact angle

The water contact angles are plotted in Fig. 4c for samples containing from 0 to 40 wt% TiO_2_. The ABS/TiO_2_ composites showed larger contact angles than the pure ABS fibers. As the concentration of TiO_2_ increases, the water contact angle of the self-cleaning film shows an increasing trend which means excessive TiO_2_ concentration will reduce the hydrophilicity of the self-cleaning film^56^.

### Photo-catalysis study

Figure 4d shows the UV absorbance of nanometer TiO_2_ with different crystalline types. As can be seen, the wavelength of light absorption is mainly in the ultraviolet region, and a small amount extends to the visible range. The photocatalytic properties of TiO_2_ allow a small amount of band-gap light to be used effectively for molecular decomposition. The absorption edge positions obtained by using the tangent method are as follows: Anatase-395 nm, P25-403 nm, Rutile-425 nm. According to the formula (Eq. 1), the bandgap energy of the three materials is calculated: Anatase-3.14 eV, P25-3.08 eV, Rutile-2.92 eV.

1 $${\text{Eg}}\;({\text{eV}})=1240{\text{/Absorption}}\;{\text{Edge}}\;({\text{nm}})$$

The bandgap energy of Anatase is the largest, followed by P25, and Rutile is the smallest. For its band energy, the greater the band energy, the stronger the reducibility of photo-generated electrons and the stronger the oxidability of holes, but the visible light absorption range will be reduced. However, too small Eg will cause more photogenerated electron–hole pairs to recombine without migrating to the surface of the catalyst, thereby affecting the activity of the catalyst.

The photocatalyst activity was assessed by calculating the dye decomposition rate as:

2 $$\upeta = \frac{{{\text{A}}_{0} - {\text{A}}_{{\text{t}}} }}{{{\text{A}}_{0} }} \times 100$$

where A_0_ (mg/L) is the initial dye concentration and A_t_ (mg/L) is the concentration after decomposition.

**Figure 5:**
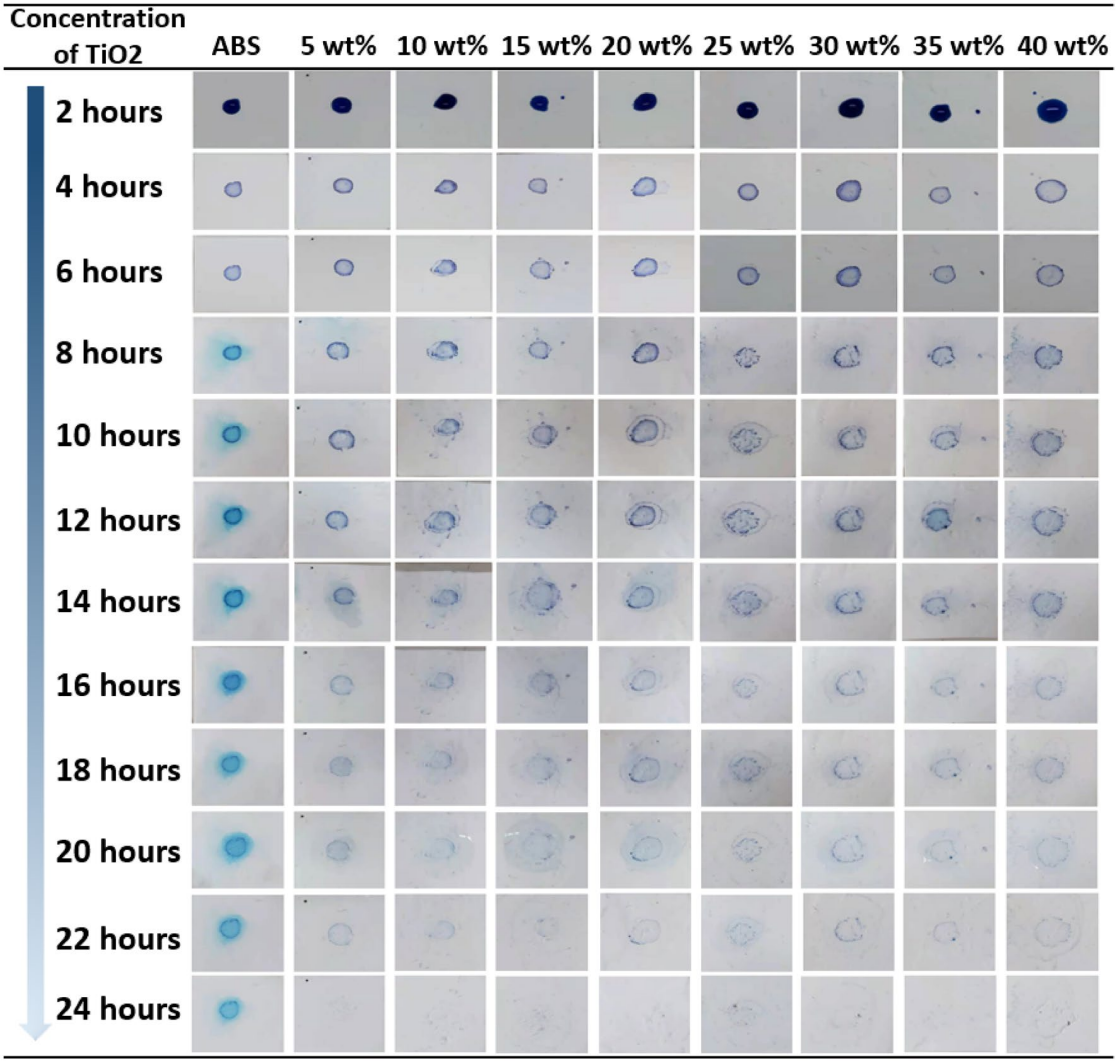
First cycle photographic images (Contamination point 1) showing the discoloration of methylene blue on SCF specimens in an inside-door environment over 24 h.

To investigate the self-cleaning capabilities of the SCFs, photocatalytic trials were carried out using UV-A, UV-B and UV-C lamps (Fig. 4e). The pure ABS film showed no catalytic decomposition of the dye, while decomposition was observed in the case of samples with TiO_2_, indicating that these films were able to remove the methylene blue. Increasing the TiO_2_ concentration was found to promote decomposition, although this effect became weaker above 20 wt%. The decomposition rates obtained from a 20 wt% TiO_2_ SCF were 45.01%, 42.00% and 36.18% under UV-A, UV-C and UV-B radiation. As noted, raising the TiO_2_ concentration increased the fiber diameter, meaning that some of the TiO_2_ could have been covered by fibers and so was not active. The decomposition rate may also have increased more slowly above 20 wt% because the relative fiber surface areas that provided catalytic sites were decreased. The above contact angle and photocatalytic reaction data suggested that these films should exhibit self-cleaning, and this was verified by a one-week dye decomposition experiment. In this trial, 0.5 ml of a 0.1 wt% aqueous methylene blue solution was applied to the surfaces of nine ABS/TiO_2_ composite films containing from 0 to 40 wt% TiO_2_ and these samples were placed outdoors for one week. Video S2 shows the results obtained from the sample containing 25 wt% TiO_2_. The images in Fig. 5 confirm that the dye on these SCFs faded from its original blue color, indicating a positive decomposition effect. In comparison with other means of fabricating SCFs, such as powder film formation^57^, sol–gel processes^58^, chemical vapor deposition^59^ and physical vapor deposition^60^, the proposed SCF preparation process is simpler and efficient and the present SCFs also showed effective degradation of an organic compound (that is, methylene blue)^61–63^.

### Recyclable performance evaluation

**Figure 6:**
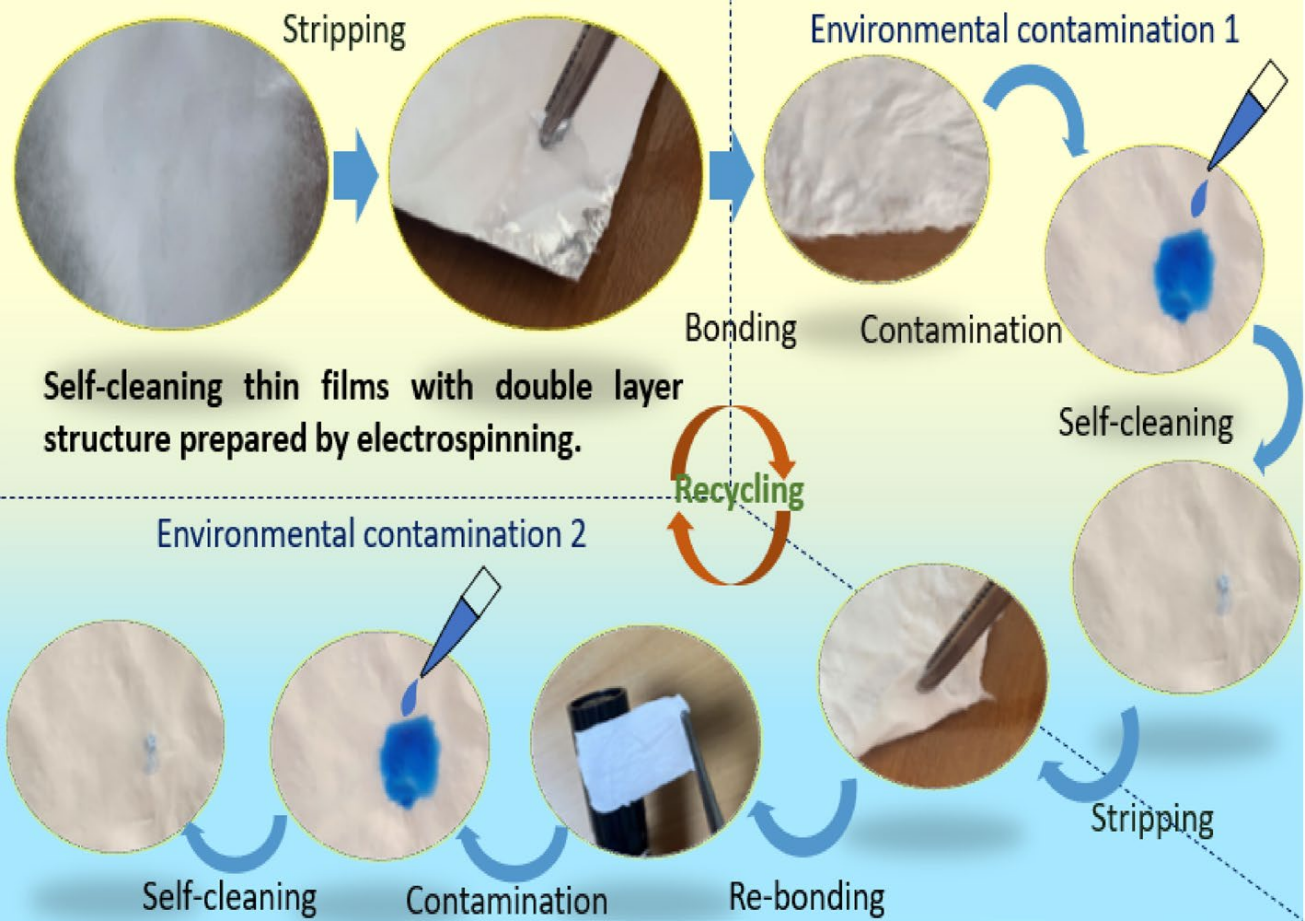
Photographic images showing the concept of a bilayer TiO_2_-based photocatalytic SCF.

**Figure 7:**
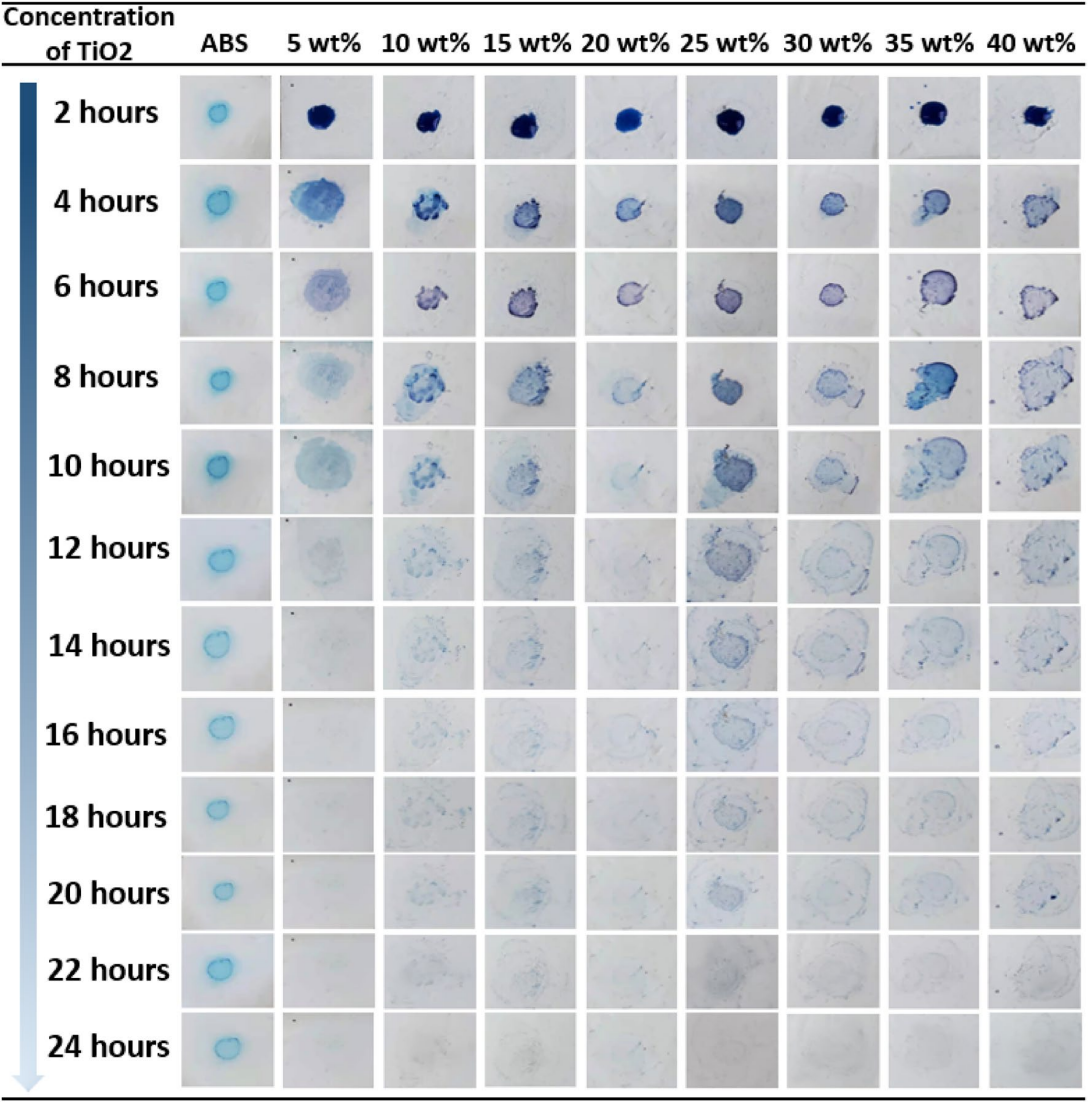
Second cycle photographic images (Contamination point 2) showing the discoloration of methylene blue on SCF specimens in an inside-door environment over 24 h.

Figure 6 presents a series of images summarizing the concept of our recyclable SCF, which can be readily applied and removed from different surfaces. This type of SCF could be installed in various environments to realize the removal of pollutants and subsequently removed and reused. In order to prove the recyclable characteristics of the proposed double-layer self-cleaning film, we used the same batch of samples to carry out a second self-cleaning characteristic experiment (pollution point 2) since the first self-cleaning effect experiment (pollution point 1). Figure 7 shows the experimental process and results of the second cycle methylene blue fading (self-cleaning). Pure ABS film still cannot fade methylene blue, which means no self-cleaning effect. The composite fiber film containing TiO_2_ all completed the cleaning of pollutants within 24 h, and the time required was the same as the first experiment. This confirms that the proposed film can be self-cleaning of pollutants multiple times, and supports the recyclable characteristics. In addition, the methylene blue fading process induced by the self-cleaning films of different concentrations of TiO_2_ is relatively uniform, maintaining positive consistency with the results of the first experiment (Contamination point 1), again proving that the TiO_2_ in the film maintains its inherent photocatalytic ability.

**Figure 8:**
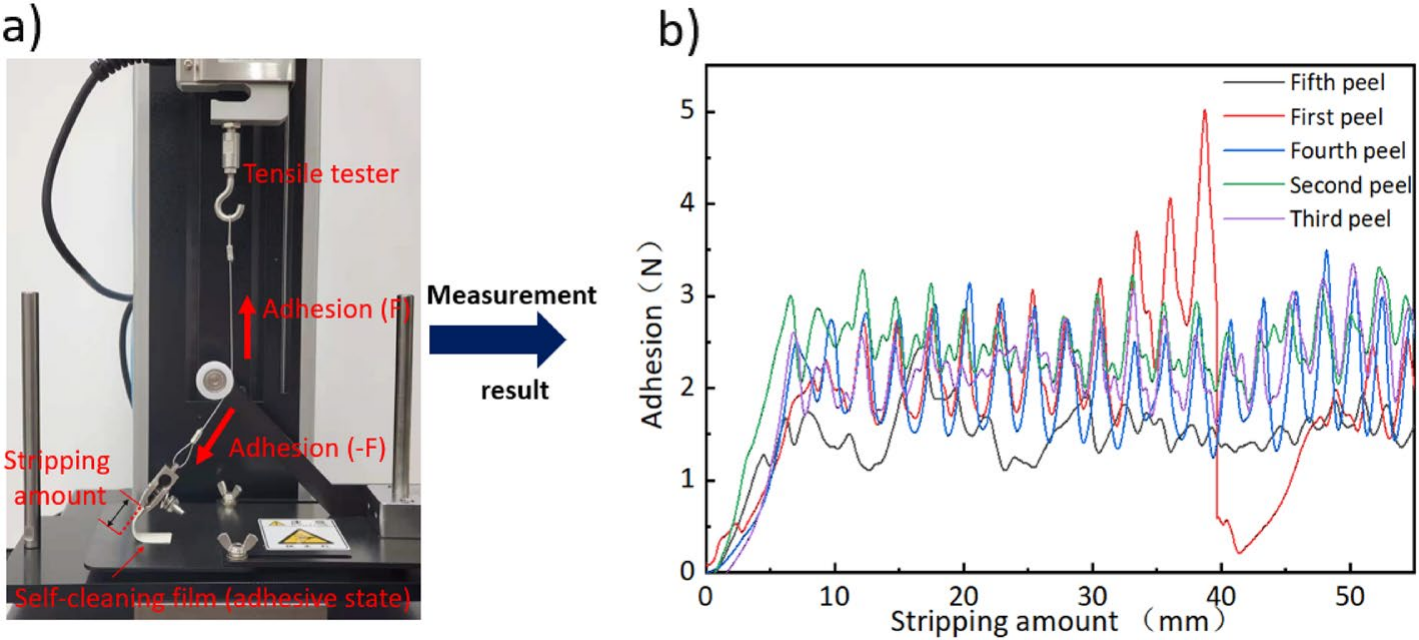
(**a**) Self-cleaning film adhesion and strength measurement method. (**b**) Film adhesion measurement results after five times of adhesion and peeling.

In the case of recycling, the self-cleaning film needs to be adhered or peeled off to different pollution points multiple times, so the adhesion and strength need to be guaranteed. Figure 8 shows the adhesion test method and experimental results. Using a tensile tester and a specific pulley block device can restore the true state of the film when it is peeled off. Additionally, we could simultaneously evaluate the adhesion and strength. As shown in Fig. 8a, when the film is gradually peeled off, with the gradual rise of the tensile tester, the peeled part is subjected to the tensile force of the steel wire rope, which is equal to the adhesion force. Figure 8b shows the experimental results of the same thin film being adhered and peeled off 5 times. With the increase in the number of installation and removal, its adhesion has declined very slightly, but it is still stable near 2 N. As a material that provides viscosity, PVA film will suffer a certain loss in the process of continuous adhesion and peeling, but it still maintains the basic viscosity and strength, supporting the film's recyclable function.

**Figure 9:**
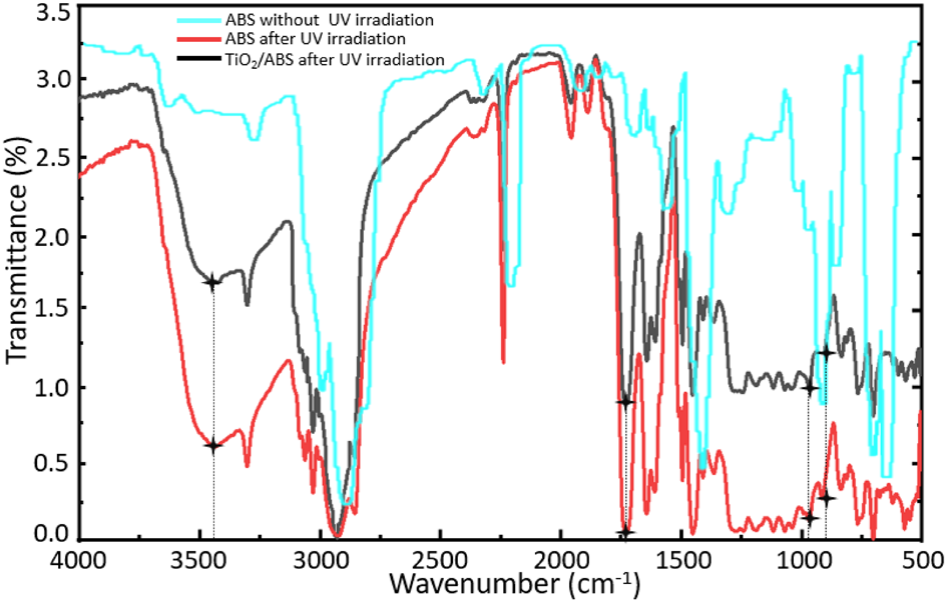
FTIR spectra of an ABS film without UV irradiation, an ABS film after UV irradiation for 200 h and a TiO_2_/ABS film after UV irradiation for 200 h.

In the absence or after the depletion of UV stabilizers, the poly(butadiene) (PB) phase in ABS may undergo photo-oxidative degradation, leading to mechanical failure of the films. This process involves photolysis of the trans-methylene bonds, after which the resulting free radicals are oxidized to generate carbonyl and hydroxyl products^64^. It was considered that this effect of UV light on the ABS might be mitigated to some extent after adding the TiO_2_ based on the absorption of UV light by the oxide^65^, and so photo-degradation experiments were conducted. In these trials, ABS and ABS/TiO_2_ films were exposed to UV light for 200 h at an intensity of 5.12 μW cm^−2^ and a wavelength of 352 nm. FTIR spectra were subsequently acquired to assess chemical changes in the ABS microstructure. In Fig. 9, the carbonyl and hydroxyl peaks at 1721 and 3465 cm^−1^, show significant variations that suggest a change in chemical structure associated with the oxide. The spectrum after UV exposure also shows significant changes in the absorption bands at 966.92 and 911.43 cm^−1^ that correspond to the trans vinyl groups in PB and 1,2-butadiene, respectively. These modifications confirm a change in the PB microstructure, attributed to chain breaking and cross-linking. From a comparison of the two films, it is apparent that the sample containing TiO_2_ exhibited less change, and so this oxide appears to mitigate photodegradation of the ABS.

## Conclusions

Recyclable SCFs having a bilayer structure were successfully prepared. Microscopic characterization and a series of performance tests verified that these films exhibited improved service life, efficient self-cleaning, recyclability and suitable mechanical strength. The good adhesion of the PVA component allowed these SCFs to be reused, which would be helpful in practical applications. Interestingly, studies have shown that TiO_2_ also has the potential to inactivate the COVID-19 virus^66^. The bilayer structure of these films could allow the fabrication of medical products that can be recycled, thus reducing costs and waste.

## Supplementary Information

Supplementary Video S1.

Supplementary Video S2.

Supplementary Legends.
