# Supplementary material for: Electro-spray deposited TiO2 bilayer films and their recyclable photocatalytic self-cleaning strategy
Source: Sci Rep. 2022 Jan 28;12:1582. doi: 10.1038/s41598-022-05633-w (PMC8799740; doi:10.1038/s41598-022-05633-w)
Supplement: Supplementary file 3 — Supplementary Legends. [file 41598_2022_5633_MOESM3_ESM.docx]

Supporting Information

**Electro-spray Deposited TiO_2_ Bilayer Films: A Recyclable Photocatalytic Self-Cleaning Strategy**

Kewei Song^1^, Yue Cui^1^, Liang Liu^1^, Boyang Chen^1^, Kayo Hirose^2^, [Md. Shahiduzzaman](https://www.sciencedirect.com/science/article/abs/pii/S1385894721000607#!)^3,^*, Shinjiro Umezu^1,4^*

*^1^Graduate School of Creative Science and Engineering, Department of Modern Mechanical Engineering, Waseda University, 3-4-1 Okubo, Shinjuku-ku, Tokyo 169-8555, Japan*

*^2^Anesthesiology and Pain Relief Center, The University of Tokyo Hospital, 7-3-1 Hongo, Bunkyo-ku, Tokyo 113-8655, Japan*

*^3^Nanomaterials Research Institute, Kanazawa University, Kakuma, Kanazawa 920-1192, Japan*

*^4^Department of Modern Mechanical Engineering, Waseda University, 3-4-1 Okubo, Shinjuku-ku, Tokyo 169-8555, Japan*

*Corresponding author (s): [shahiduzzaman@se.kanazawa-u.ac.jp](mailto:shahiduzzaman@se.kanazawa-u.ac.jp); [umeshin@waseda.jp](mailto:umeshin@waseda.jp)

**Video-S1:** Process for the preparation of proposed bilayer structured TiO_2_-based photocatalytic SCFs by electrostatic spinning.

**Video-S2:** Self-cleaning process of the proposed bilayer structured TiO_2_-based photocatalytic self-cleaning films.
